# Triggering of Polymer-Degrading Enzymes from Layered Double Hydroxides for Recycling Strategies

**DOI:** 10.3390/ijms24010831

**Published:** 2023-01-03

**Authors:** Angela Romano, Antonella Rosato, Stefano Bianchi, Giulio Zanaroli, Annamaria Celli, Grazia Totaro, Laura Sisti

**Affiliations:** Dipartimento di Ingegneria Civile, Chimica, Ambientale e dei Materiali, Università di Bologna, Via Terracini 28, 40131 Bologna, Italy

**Keywords:** polymer-degrading enzyme, enzyme triggering, multilayer recycling

## Abstract

The use of degrading enzymes in polymer formulation is a very attractive strategy to manage the end-of-life of plastics. However, high temperatures cause the denaturation of enzymes and the loss of their catalytic activity; therefore, protection strategies are necessary. Once protected, the enzyme needs to be released in appropriate media to exert its catalytic activity. A successful protection strategy involves the use of layered double hydroxides: cutinase, selected as a highly degrading polyester hydrolytic enzyme, is thermally protected by immobilization in Mg/Al layered double hydroxide structures. Different triggering media are here evaluated in order to find the best releasing conditions of cutinase from LDH. In detail, phosphate and citrate–phosphate buffers, potassium carbonate, sodium chloride, and sodium sulfate solutions are studied. After the comparison of all media in terms of protein release and activity retained, phosphate buffer is selected as the best candidate for the release of cutinase from LDH, and the effect of pH and concentration is also evaluated. The amount of the enzyme released is determined with the Lowry method. Activity tests are performed via spectrophotometry.

## 1. Introduction

The use of degrading enzymes in polymer formulations is an attractive strategy for the end-of-life of plastics. A critical issue, however, is their thermal lability: the protection of enzymes is a necessary step to implement their use during polymer processing where high temperatures, which cause the denaturation of enzymes and the loss of their catalytic activity, are commonly employed. Among several enzymes, cutinase is a highly degrading polyester hydrolytic enzyme: biopolymers such as poly(butylene succinate) (PBS), poly(butylene succinate-*co*-adipate) (PBSA), and poly(caprolactone) are successfully degraded by cutinase [1,2,3]. The immobilization of cutinase in inorganic host structures such as layered double hydroxides (LDH) has been demonstrated as an effective approach to thermally protect the enzyme [4]. LDH, whose general formula is

[M^II^_1−x_ M^III^_x_ (OH)_2_]^x^ + (A^n−^)_x/n_ mH_2_O

wherein M^II^, M^III^, and A^n−^ denote divalent cations (Mg^2+^, Ca^2+^, Zn^2+^, Cu^2+^, Co^2+^, Ni^2+^, etc.), trivalent cations (Al^3+^, Fe^3+^, Ga^3+^, and Cr^3+^), and an interlayer anion (Cl^−^, CO_3_^2−^, NO_3_^−^, etc.) [5,6], are suitable for enzyme immobilization [7,8,9,10,11,12,13,14,15]. Once protected, the enzyme needs to be released in appropriate media to exert its catalytic activity. When the enzyme is immobilized on LDH, it remains dormant. The triggering occurs through an ion exchange mechanism in salt solutions: the anions from the salt interact with the positively charged lamellae of the host structure and substitute the anionic protein, which can be released and recover its degrading activity. Such a strategy has a really high potential, since the system developed could enhance the biodegradation rate of biopolymers, for example, or it could be used in a polymeric adhesive formulation for multilayers, allowing the degradation of the adhesive at the end-of-life of the material, with consequent delamination and recycling of layers, which are now poorly recyclable. As a matter of fact, the approach here proposed is a recycling strategy by design.

The first release experiments of several Mg/Al LDH/cutinase, containing different amounts of enzyme, have been previously reported [4]: the findings demonstrated that the best option in terms of protein recovered and immobilization efficiency is the sample containing a 2/1 ratio of cutinase with respect to Al; therefore, in the current study, the best triggering conditions are evaluated in terms of medium, pH, and concentration.

In general, the release follows two steps. First, a rapid process, and then a maintained, slow release, related to the anionic exchange with anions from the medium [16]. The anion exchange depends on the charges and the ionic radius, meaning that with the increase in charges and decrease in ionic radius, the exchangeability of the incoming anion increases. For example, the order for simple inorganic anions decreases as follows: CO_3_^2−^ > HPO_4_^2−^ > SO_4_^2−^ for divalent anions, and OH^−^ > F^−^ > Cl^−^ > Br^−^ > NO_3_^−^ > I^−^ for monovalent anions [17,18]. 

Several studies have reported on the release of molecules from LDH. For example, Ralla et al. [19] reported the adsorption and separation of model proteins (such as hemoglobin and human serum albumin) from MgAl/LDH in several buffers. They stated that if a release is requested, intercalation should be avoided in favor of adsorption, because in the case of intercalation, the release would be difficult. Thus, the presence of intercalated carbonate actually enables a reversible binding of adsorbed proteins. The authors tested different concentrations of carbonate solution. Moreover, they clarified that the largest amount of protein could be desorbed when applying pH values beneath the protein’s isoelectric point to decrease electrostatic interactions between the protein and the LDH surface. They observed a change in protein elution as a function of pH, but no significant differences by changing the concentrations of buffers. Concerning the media, the authors evaluated carbonate (500 mM at pH 10) or phosphate buffers (50 mM at pH 7 and 12), acetate (50 mM at pH 5), as well as buffer additives (i.e., 20% *v/v* in 50 mM buffer) such as polyethylene glycol or glycerol [19]. 

Potassium carbonate is reported also for the de-intercalation of amino acids and drugs [16,20], while phosphate buffer is employed also for the release of nisin from the LDH structure [11]. The authors observed that the release process could be affected by the concentration and is mainly based on the anion exchange between nisin molecules and phosphate anions. 

Cellulase and papain are reported to be released from a MgAl/LDH in aqueous sodium sulfate solution (1.2 wt%) or saturated sodium sulfate solution [8,9]. Desorption assays were performed for a Lipase immobilized on ZnAl/LDH-Cl in sodium chloride (NaCl) 1–2 M or 3% *v/v* Triton X-100 (a common non-ionic surfactant and emulsifier based on polyethylene glycol tert-octylphenyl ether) [21]. NaCl is also reported for the release of antibiotics [22].

In view of the above considerations, phosphate and citrate–phosphate buffers, potassium carbonate, sodium sulfate, and sodium chloride were chosen as release media. Besides the already mentioned affinity of phosphate for the LDH structure, the phosphate buffer is also the optimal medium for the activity of cutinase toward the degradation of polyesters [1]. Its basic pH (8) is near the isoelectric point of cutinase (i.e., 7.8), thus no charges should be present, and the detachment of the enzyme is facilitated [19]. For the same reasons, the citrate–phosphate buffer at pH 8 was tested as well. 

All media were first tested in terms of the degradation ability of the free enzyme against poly(butylene succinate-*co*-adipate) (PBSA), as a polyester reference, through film weight-loss tests. Then, fast-release tests (4 h) and time-release profiles were carried out for cutinase immobilized on LDH. After the comparison of all media, phosphate buffer was selected as the best candidate for the release of cutinase from LDH, and the effect of pH and concentration was also assessed. The amount of enzyme released from the LDH is determined via the measurement of the protein concentration with the Lowry method. Activity tests are performed via spectrophotometry.

## 2. Experimental

### 2.1. Materials

Aluminum nitrate Al(NO_3_)_3_∙9H_2_O, magnesium nitrate Mg(NO_3_)_2_∙6H_2_O, NaOH, sodium carbonate Na_2_CO_3_, and sodium bicarbonate NaHCO_3_ were purchased from Aldrich Chemicals. Potassium carbonate (K_2_CO_3_), sodium chloride (NaCl), and sodium sulfate (Na_2_SO_4_) were purchased from Carlo Erba Reagents. Cutinase from *Humicola insolens* was provided by ChiralVision (NZ 51032). Sodium phosphate dibasic (Na_2_HPO_4_), sodium phosphate monobasic (NaH_2_PO_4_), citric acid (C_6_H_8_O_7_), 4-nitrophenyl butyrate (4-NPB, ≥98%), copper sulfate (CuSO_4_), bovine serum albumin (BSA), sodium tartrate (C_4_H_4_Na_2_O_6_), and 2N Folin and Ciocalteu’s phenol reagent were purchased from Merck. A commercial co-polyester, namely poly(butylene succinate-*co*-adipate) (PBSA, commercial name bioPBS FD92PM), having the following composition, (PBS)_0_._7_-(PBA)_0_._3_, was supplied by PTT MCC Biochem Company, Bangkok, Thailand. All the materials were used as received.

### 2.2. Film Weight Loss

Thin PBSA films (1 cm^2^, 0.15 mm thickness) were incubated in the different triggering solutions (2 mL) containing 10 U/mL of free cutinase. The incubation was performed in 5 mL screw-cap glass vials in a thermostatic bath (40 °C) under gentle mixing. Controls without cutinase were also set up under the same conditions. After 15, 30, and 60 min of incubation, sacrificial PBSA films were washed with deionized water, dried overnight under a vacuum, and then weighed. The weight loss was calculated by subtracting the dry weight remaining at a specific sampling time from the initial one. The enzymatic degradation rate was calculated as mg_PBSA_/h/cm^2^.

### 2.3. Immobilization of Cutinase on LDH

The LDH was prepared according to the procedure reported by Romano et al. [4]. Briefly, Mg(NO_3_)_2_·6H_2_O (0.209 g, 8.16 × 10^−4^ mol) and Al(NO_3_)_3_·9H_2_O (0.153 g, 4.08 × 10^−4^ mol) were mixed with deionized and decarbonated water (50 mL) and added dropwise to a water solution (100 mL) containing cutinase (5.6 mL, 20 mg/mL, corresponding to 0.102 g and 8.36 × 10^−4^ mol of enzyme). The molar ratio enzyme/Al^3+^, evaluated from the aminoacidic sequence of cutinase (average molecular weight 122 g/mol), was 2/1. The coprecipitation was carried out over 3 h, with stirring, under nitrogen flow. The pH was kept constant (10 ± 0.4) with the addition of NaOH solution (0.049 M). After the coprecipitation, the reaction mixture was aged for 3 h at room temperature (RT). The solid product was filtrated on Büchner, washed with 300 mL of deionized and decarbonated water, and dried overnight in a vacuum oven at RT. The final reaction medium and the washing solution were collected to measure the amount and activity of the residual enzyme and the immobilization efficiency was 95%. The enzyme amount was also evaluated in terms of the ratio between the amount of immobilized enzyme with respect to the LDH/enzyme mass obtained (w_cutinase_/w_LDH/Cut_ 0.52 mg/mg), in order to have an idea of the absolute amount of enzyme present, independent of the immobilization efficiency. LDH is labeled LDH/Cut-2/1. A reference sample containing carbonate was similarly prepared. Mg(NO_3_)_2_·6H_2_O (0.295 g, 1.15 × 10^−3^ mol) and Al(NO_3_)_3_·9H_2_O, (0.215 g, 5.75 × 10^−4^ mol) were mixed with deionized water (50 mL) and added dropwise to a water solution (100 mL) containing Na_2_CO_3_ (0.19 g, 1.79 × 10^−3^ mol) and NaHCO_3_ (0.065 g, 7.74 × 10^−4^ mol). The reaction was carried out over 3 h, with stirring, at RT. The pH was kept constant (10 ± 0.5) with the addition of NaOH solution (0.07 M). After the coprecipitation, the reaction mixture was aged for 3 h at RT. The solid product, labeled LDH/CO_3_, was filtrated, washed with 300 mL of deionized water, and dried at RT in a dynamic vacuum oven.

### 2.4. Triggering of Enzyme from LDH

An amount of 5 mg of LDH was put into 1 mL of different solutions (see Table 1). After 4 h, the suspensions were centrifuged (12,000 rpm, RT, 2 min) and the supernatants were recovered for the measurements of the enzyme amount released and their relative activity. In the case of the release kinetics studies, 50 mg of LDH was put into 10 mL of different solutions. At specific times, suspensions were centrifuged (5000 rpm, RT, 5 min) and a small aliquot of the supernatants was recovered for the measurements of the enzyme amount released and their relative activity. Kinetics experiments were performed over 12 h on selected media.

The experiments were carried out on two LDH/Cut samples with high reproducibility of the results.

The effect of buffer concentration and pH on the enzyme release was also verified. In the first case, two concentrations were tested, namely 0.3 and 0.5 M, and compared to the results obtained for sodium phosphate buffer, 0.1 M. The pH was maintained at 8. In the second case, pH 4 and 6 were tested and compared to the results obtained for sodium phosphate buffer pH 8. The concentration was fixed at 0.1 M. 

### 2.5. Characterization

All LDHs were prepared in an automatic system reactor equipped with a pH-meter Knick 765 with an XS polymer PlusPro electrode, two peristaltic pumps, Reglo Digital MS2/08, for metals, and NaOH solutions, controlled by LABWORLDSOFT V5 IKA software.

The infrared spectra of sample powders were recorded on the wavenumber range 650–4000 cm^−1^ using a Perkin Elmer Spectrum One FT-IR spectrometer equipped with a Universal ATR sampling accessory. For each spectrum, 16 scans were taken at a resolution of 2 cm^−1^. 

The X-ray diffraction (XRD) analysis was carried out at room temperature by means of a Philips X-Pert Pro diffractometer. Data were acquired by exposing the samples to Cu-Kα X-ray radiation. The patterns were collected with a step size of 0.03°, over a 2θ range of 2.0–80°, with an accumulation time of 10 s per step.

Thermogravimetric analysis (TGA) was performed under an air atmosphere for all samples, using a PerkinElmer TGA7 apparatus (gas flow 30 mL min^−1^) at a 10 °C min^−1^ heating rate from 50 to 800 °C. The T_20_^D^ values, i.e., the temperatures at which samples lose 20% of their mass, were measured. To test cutinase, a lyophilized sample was used, obtained through freeze-drying in a skim milk solution. Samples were kept overnight under a vacuum at RT before analysis.

The amount of enzyme was determined by the Lowry method. Each sample (0.5 mL) was boiled for 1 min, rapidly cooled on ice, and supplemented with 2.5 mL of a reagent mixture consisting of 2% Na_2_CO_3_ (in NaOH 0.1 N), 1% CuSO_4_ (in dionex water), and 2% C_4_H_4_Na_2_O_6_ (in dionex water) in a 100:1:1 ratio. After the reagent addition, the samples were immediately mixed and incubated for 10 min. A volume of 0.25 mL of Folin and Ciocalteu’s phenol reagent (1 N) was then added to each sample, which was immediately vortexed. After 30 min of incubation in the dark at room temperature, the absorbance of each sample was then measured at 540 nm by means of a UV-Vis spectrophotometer (Varian Cary 100 bio, Dual Beam). The enzyme concentration was calculated through a calibration curve prepared using BSA dilution series (1 to 50 mg/L of BSA) as standards; the calibration curve was constantly verified.

The enzyme activity was simultaneously checked by a continuous spectrophotometric assay using 4-nitrophenyl butyrate (4-NPB) as substrate. A volume of 0.1 mL of each sample was added to 3 mL of sodium–potassium phosphate buffer (0.1 M, pH 7.8) containing 1 mM of 4-NPB. In the presence of the enzyme, this substrate is rapidly hydrolyzed into butyric acid and 4-nitrophenol, whose formation can be spectrophotometrically measured at 420 nm over time. In particular, the absorbance (Abs 420 nm) was continuously monitored until constant using a UV-Vis spectrophotometer set at 25 °C, and the absorbance per minute was obtained using the maximum linear rate. One unit of enzyme is defined as the amount of enzyme that hydrolyses 1 μmol of 4-nitrophenyl butyrate to butyric acid and 4-nitrophenol per minute under the assay conditions. 

## 3. Results and Discussion

### 3.1. Effect of Medium on the Release of Enzyme from the LDH Structure

The release of molecules from LDH is based on the anions’ affinity for the cationic structure of the inorganic layers, and therefore on their exchangeability within the interlayer space, hosting the anions. This exchangeability increases by increasing the charge of the incoming anion and by decreasing its ionic radius [17]. Moreover, when bio-active compounds are involved, interfacial interaction between the hosting structure and the guest molecule, i.e., adsorption on either the outer surface or within the layers, occurs [23]; therefore, exchanging anions can also substitute the enzyme through surface interactions. Zou and Planck [8,9] reported the use of Na_2_SO_4_ 0.08 M for the release of cellulase from LDH. Therefore, preliminary kinetics studies (up to 12 h) were carried out on MgAl/cutinase-based LDH in sulfate solution and water, and the findings were reported by Romano et al. [4]. Briefly, most of the cutinase was released in Na_2_SO_4_ in the first 4 h, and after this time, the release profile tended to reach a plateau. Moreover, the recovered enzyme was completely active, indicating that cutinase mostly returns to its original conformation and activity. On the other hand, the release of cutinase in water was negligible, reaching 10% in 4 h. After 12 h, the total activity recovered was 67% in sulfate solution and 14% in water. Moreover, kinetic release studies in Na_2_SO_4_ 0.08 M on several LDH/Cut samples with different loadings of cutinase (ranging from 3.4/1 to 0.5/1 in terms of molar ratio with respect to Al^3+^), demonstrated that the 2/1 loading is the optimum balance in terms of immobilization efficiency (80%) and higher protein release (60%), without a loss of activity in the enzyme recovered [4]. 

Along with Na_2_SO_4_, other salt solutions were here tested for the release of cutinase from LDH/Cut-2/1—phosphate buffer, citrate–phosphate buffer, potassium carbonate, and sodium chloride (Table 1)—in order to evaluate the effect of different exchanging anions. 

Phosphate buffer was chosen since it is the optimal medium for the activity of cutinase toward the degradation of polyesters commonly used as tie-layers [1], and phosphate anion is reported to have a high affinity for the LDH structure [24]. Moreover, the pH 8 of the buffer is near the isoelectric point of cutinase (i.e., 7.8), thus no charges should be present, favoring the detachment of the enzyme [19]. For the same reasons, the buffer citrate–phosphate at pH 8 was tested as well. Potassium carbonate was chosen because of the highest affinity of CO_3_^2−^ for LDH structures. It is indeed reported for the de-intercalation of amino acids and drugs [16,20]. The concentration of these solutions was kept around 0.1 M. Sodium chloride is reported for the desorption of *Pseudomonas cepacia* lipase [21] and for the release of antibiotics [22], thus it has been taken into consideration. However, the concentration of the NaCl solution tested was 1 M. Indeed, the chloride ion has one negative charge compared to the other anions tested, so it was necessary to increase its concentration to force the exchange with the enzyme. 

First, the ability of the free enzyme to degrade PBSA in such solvents was tested via film weight-loss tests. The enzymatic degradation rate was calculated as mg_PBSA_/h/cm^2^. Moreover, the activity of cutinase in the same solutions was measured through a continuous spectrophotometric method using *p*-nitrophenyl butyrate (*p*-NPB) as substrate.

**Figure 1:**
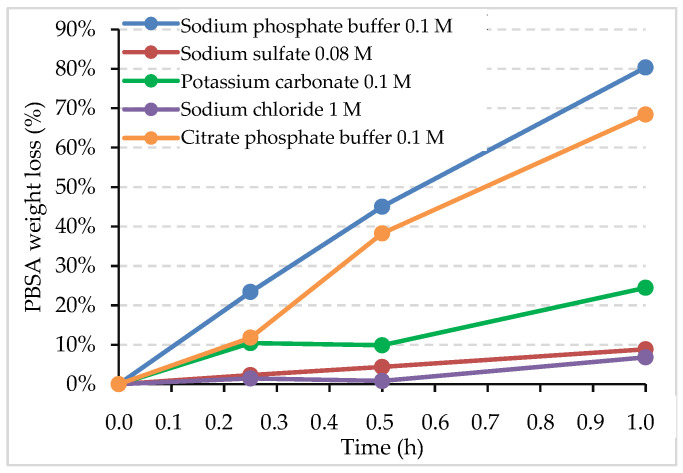
Weight loss (%) of PBSA by cutinase in the different triggering solutions as a function of the incubation time (hours).

No degradation was observed in the enzyme-free controls (data not shown). Conversely, cutinase displayed the highest degradation activity in sodium phosphate buffer 0.1 M, followed by citrate–phosphate buffer 0.1 M, and potassium carbonate (Figure 1). The degradation rates (mg/h/cm^2^) are reported in Table 2, as well as the activity of cutinase in the different triggering solutions, measured on *p*-NPB. One unit of enzyme is defined as the amount of enzyme that hydrolyzes 1 µmol of *p*-NPB to butyric acid and 4-nitrophenol per minute. Cutinase exhibited the highest hydrolytic activity in sodium phosphate buffer and citrate phosphate buffer, followed by sodium sulfate solution (Table 2). Data on potassium carbonate are not available since the negative control (*p*-NBP without enzyme in K_2_CO_3_ solution) had a colorimetric response. Therefore, the enzymatic assay of cutinase on *p*-NPB confirmed that sodium phosphate buffer can be selected as the best solution for the triggering and release of cutinase from the LDH structure.

**Figure 2:**
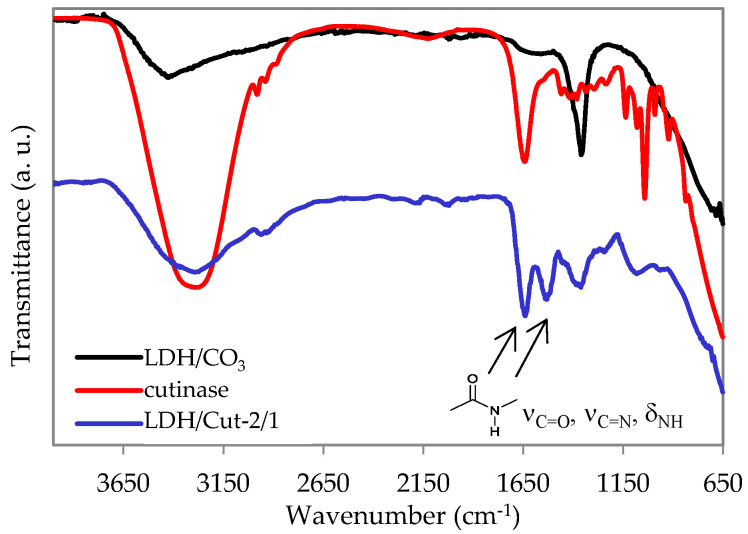
ATR FT-IR curves of LDH/Cut-2/1, cutinase, and LDH/CO_3_ as a reference sample.

**Figure 3:**
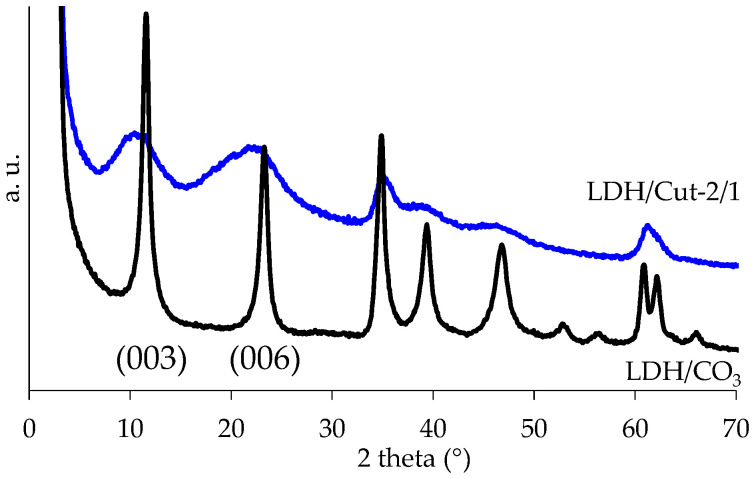
X-ray diffraction patterns of LDH/Cut-2/1, and LDH/CO_3_ as a reference sample.

**Figure 4:**
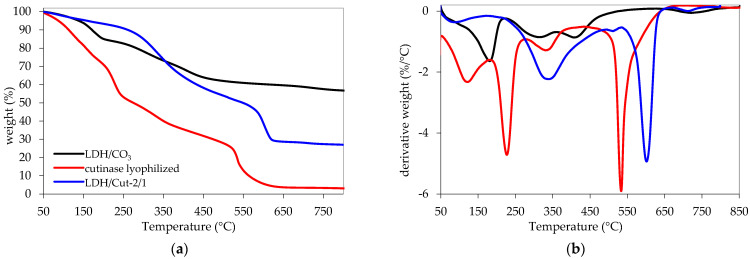
(**a**) TGA and (**b**) dTGA curves of LDH/Cut-2/1, cutinase, and LDH/CO_3_ as a reference sample.

The triggering tests in all the above-mentioned salt solutions were performed on a sample prepared by immobilizing cutinase on an Mg/Al LDH structure through a coprecipitation method. The sample was fully characterized by FT-IR, XRD, and TGA and the results are are shown in Figure 2, Figure 3 and Figure 4. Briefly, the presence of cutinase in the LDH structure is confirmed by the amide I peak, appearing at 1640 cm^−1^, which is mainly due to the C=O stretching vibrations, together with a sharp amide II band at 1530 cm^−1^, due to the bending modes of the N–H group and the stretching mode of the C–N group. A wide absorption band appears at 3300 cm^−1^, which is due to the NH and OH stretching of the hydroxyl group and water molecules present in the interlayer space of LDH. Moreover, the LDH spectrum shows the characteristic lattice vibration bands at low regions (ν_M-O_ and δ_O-M-O_). The carbonate stretching at 1360 cm^−1^ is also visible, highlighting its partial co-intercalation (Figure 2). The immobilization of cutinase is also highlighted by the X-ray diffraction pattern (Figure 3): large halos are visible and the maxima of (003) and (006) reflections are shifted to slightly lower 2*θ* values with respect to the reference sample, indicating both the presence of carbonate and cutinase mostly on the surface of lamellae, as already reported [4]. Immobilization is preferred with respect to intercalation, since the release is reported to be favored [19]. Concerning the thermogravimetric experiments, the curves and data reported in Figure 4 and Table 3 also confirm the presence of cutinase, thermally stabilized by the hosting structure since the T_D_^20^ of LDH/Cut-2/1 is substantially higher with respect to free cutinase. The LDH/Cut-2/1 profile shows four losses and the protein should be lost in the range of 200–500 °C. Zou et al. [9] reported that an intercalated cellulase loses weight in the range of 400–530 °C, while Dong et al. [7] reported that immobilized *Candida* lipase loses weight from 200 to 300 °C. The profile of the reference LDH is coherent with already reported data [25]. The four steps correspond to (i) interlayer water (70–190 °C); (ii) Al(OH)_3_ (190–280 °C); (iii) Mg(OH)_2_ (280–405 °C); and (iv) CO_2_ (405–580 °C). The remaining residue is a solid solution of mixed oxides MgO and Al_2_O_3_. 

Such findings highlight that the LDH prepared is a mixed system, containing both the enzyme (mostly immobilized on the surface) and carbonate.

The results of the triggering tests are summarized in Table 4. Between 60% and 70% of the enzyme is recovered from the LDH structure, with full activity retention of the released enzyme in most of the media. Moreover, as was observed by Romano et al. [4], 60% of the enzyme is recovered in Na_2_SO_4_. The negligible release in water is confirmed, as well as in NaCl.

Therefore, kinetics studies were carried out in buffer solutions and K_2_CO_3_. Water and sodium chloride were excluded since the release is negligible, while sodium sulfate solution was not included because its degradation rate against PBSA is quite low (0.7 mg/h/cm^2^).

**Figure 5:**
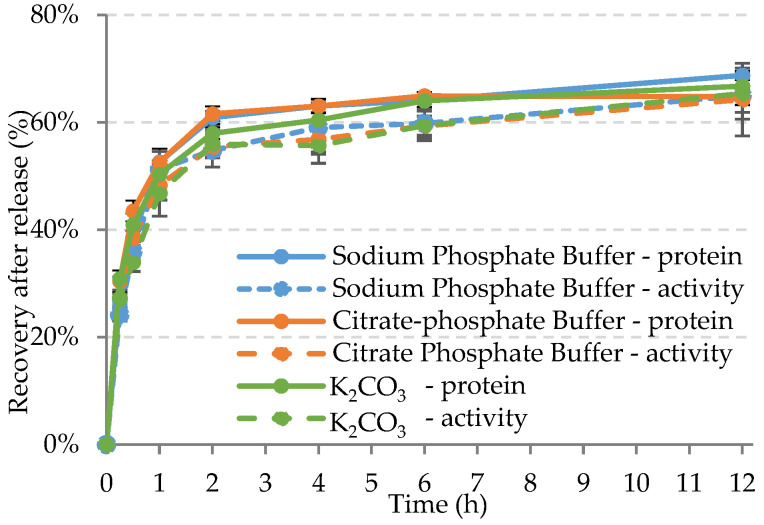
Kinetics release of cutinase from LDH/Cut-2/1 over 12 h in different solutions.

The data extrapolated at 4 h and the release profiles over 12 h are reported in Table 5 and Figure 5, respectively. As can be seen from Table 5, no significant differences are highlighted. The protein recovered is around 60%, with high retention activity (90–94%).

The release profiles (Figure 5) have the expected trend, with most of the enzyme released in the first 4 h.

In summary, the buffer solutions and K_2_CO_3_ all result in good candidates for enzyme triggering. However, sodium phosphate buffer is selected as the best option, since its degradation rate against PBSA is the highest in the conditions used in the current study (6.4 mg/h/cm^2^).

### 3.2. Effect of Sodium Phosphate Buffer Concentration and pH on the Triggering of Enzyme from the LDH Structure

Since the release is due to phosphate anions, which interact with the surface lamellae by displacing the enzyme molecules immobilized, the effect of buffer concentration on the enzyme release was tested. In particular, two concentrations were tested, namely 0.3 and 0.5 M, and compared to the results obtained for sodium phosphate buffer 0.1 M. The pH was maintained at 8. Higher concentrations were not considered, since enzyme activity could be negatively affected, as reported by Zaak et al. [26].

The results of enzyme release and its residual activity are shown in Table 6. It is evident how buffer concentration does not significantly affect the release of cutinase from the LDH structure, which is always about 60%. Moreover, the recovered enzyme is mostly active. 

Such findings agree with Ralla et al. [19]. The authors observed a change in protein elution in the function of pH, but no significant differences by changing the concentrations of buffers.

The second parameter investigated was the pH of the buffer. Indeed, a decrease in pH could reduce the interactions between cutinase and the LDH structure, favoring the release. Therefore, pH 4 and 6 were tested and compared to the results obtained for sodium phosphate buffer pH 8. The concentration was fixed at 0.1 M. As can be seen in Table 7, a decrease in pH does not significantly affect the release of cutinase from LDH, and cutinase recovers most of its initial activity (around 90%). Possibly, these experimental conditions do not determine great differences in LDH surface electrostatic interactions, whose substantial decrease would cause a further detachment of the protein.

**Figure 6:**
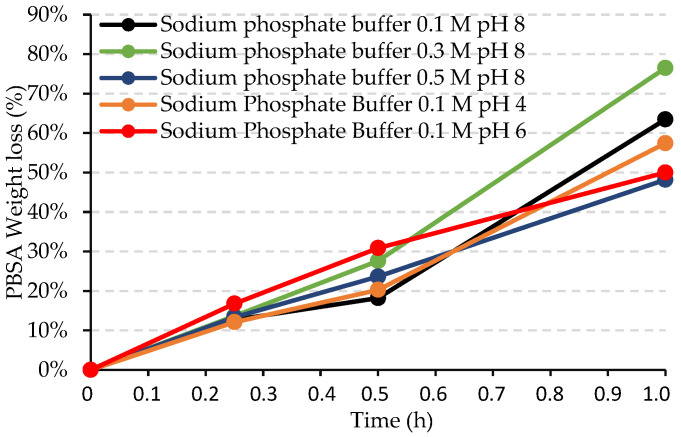
Weight loss (%) of PBSA by cutinase in phosphate buffer at different pH and concentrations as a function of the incubation time (hours).

Furthermore, the ability of the free enzyme to degrade PBSA in phosphate buffer at different concentrations and pH was checked via film weight-loss tests (Figure 6), confirming that the enzyme can degrade it in all media, with no significant changes in the rate, calculated as mg_PBSA_/h/cm^2^ (Table 8). No degradation was observed in the enzyme-free controls (data not shown).

## 4. Conclusions

The study demonstrates that the triggering of cutinase from LDH layers follows a classic mechanism: a fast release in 4 h, then a plateau is reached. The release in water or sodium chloride is negligible, while citrate phosphate buffer (0.1 M pH 8.0), sodium phosphate buffer (0.1 M pH 8.0), and potassium carbonate (0.1 M pH 11.5) presented around 60% of the protein released, with a high retention activity of around 90%. Among such media, sodium phosphate buffer is preferred since it is the best medium for cutinase degradation activity against a polyester such as PBSA. Changes in buffer concentration and pH do not affect the release of the enzyme from LDH in sodium phosphate buffer, nor the degradation rate against PBSA. These findings, together with the results already reported by Romano et al. [4], show the feasibility of including an LDH-immobilized hydrolytic enzyme in a polymer formulation by adding the immobilized enzyme during polymer processing and that, at the end-of-life of the material, the enzyme can be triggered and degrade the polymer, opening up enormous new recycling opportunities. Indeed, the biodegradation rate of bioplastics could be enhanced, the effectiveness of composting facilities improved, the oligomers and monomers could be recovered and upcycled into value-added performance materials, and multilayers could be delaminated, separated, and recovered, contributing to realizing a circular plastic economy.

## 5. Patents

Sisti L., Zanaroli G., Totaro G., Romano A., Rosato A., Celli A., Thiebes C., Fait T. Thermally stable carboxylic ester hydrolases in layered double hydroxides for intrinsic recyclable polymers. Patent Application No. 22189534.5.

## Figures and Tables

**Table 1:** Solutions tested for the release of enzymes from LDH.

Triggering Solution	Concentration (M)	pH
Sodium phosphate buffer	0.10	8.0
Citrate phosphate buffer	0.10	8.0
Sodium sulfate (Na_2_SO_4_)	0.08	7.5
Potassium carbonate (K_2_CO_3_)	0.10	11.5
Sodium chloride (NaCl)	1.00	7.4
Water (H_2_O)	-	6.5

**Table 2:** Degradation rates (mg/h/cm^2^) of cutinase on PBSA in the different triggering solutions; activity of cutinase (U/mL) in the different triggering solutions. The activity of cutinase in sodium buffer phosphate (i.e., the highest activity obtained) has been fixed as 100%. (n.a.—not available).

Triggering Solution	Degradation Rate (mg_PBSA_/h/cm^2^)	Activity (U/mL)	Relative Activity (%)
Sodium phosphate buffer	6.4	33,975 ± 531	100 ± 3
Citrate phosphate buffer	5.4	33,891 ± 1237	100 ± 7
Sodium sulfate	0.7	19,546 ± 1096	58 ± 6
Potassium carbonate	2.8	n.a	n.a
Sodium chloride	0.5	4247 ± 163	12 ± 1

**Table 3:** Thermal stability, weight loss step, and corresponding temperature range of samples.

Code	T_20_^D^ (°C) ^1^	Residue (%) ^1^	Step			
			1	2	3	4
Cutinase	153	3	28% 50–190 °C	22% 190–280 °C	18% 280–450 °C	29% 450–850 °C
LDH/CO_3_	283	56	15.9% 50–220 °C	12.6% 220–370 °C	10.5% 370–550 °C	4.7% 550–850 °C
LDH/Cut-2/1	322	27	6% 50–190 °C	42% 190–520 °C	23% 520–640 °C	2% 640–850 °C

^1^ TGA under airflow (30 mL/min); residue @ 800 °C.

**Table 4:** Protein and activity recovery of cutinase from LDH/Cut-2/1 after 4 h release in different solutions; the residual activity is calculated with respect to the protein released.

Triggering Solution	Protein Recovery (%)	Activity Recovery (%)	Retention Activity of Released Proteins (%)
Sodium phosphate buffer	59 ± 1	59 ± 2	100
Citrate phosphate buffer	69 ± 1	70 ± 1	100
Sodium sulfate	57 ± 3	59 ± 0	100
Potassium carbonate	69 ± 1	69 ± 0	100
Sodium chloride	4 ± 0	2 ± 0	50
Water	10 ± 0	10 ± 1	100

**Table 5:** Protein and activity recovery of cutinase from LDH/Cut-2/1 after 4 h release in different solutions; the retained activity is calculated with respect to the protein released.

Triggering Solution	Protein Recovery (%)	Activity Recovery (%)	Retention Activity of Released Proteins (%)
Sodium phosphate buffer	63 ± 1	59 ± 5	94
Citrate phosphate buffer	63 ± 1	57 ± 3	90
Potassium carbonate	60 ± 4	56 ± 3	92

**Table 6:** Cutinase release and residual activity in sodium phosphate buffer pH 8 at different concentrations after 4 h; the retained activity is calculated with respect to the protein released.

Concentration (M)	pH	Protein Release (%)	Activity Release (%)	Retention Activity of Released Proteins (%)
0.1	8	55 ± 4	49 ± 3	89
0.3	8	57 ± 2	47 ± 4	83
0.5	8	59 ± 1	50 ± 3	85

**Table 7:** Cutinase release and residual activity in sodium phosphate buffer 0.1 M at different pH, after 4 h. The retained activity is calculated with respect to the protein released.

Concentration (M)	pH	Protein Release (%)	Activity Release (%)	Retention Activity of Released Proteins (%)
0.1	8	55 ± 4	49 ± 3	89
0.1	6	50 ± 3	43 ± 3	86
0.1	4	52 ± 4	47 ± 3	90

**Table 8:** Degradation rates (mg/h/cm^2^) of cutinase on PBSA in phosphate buffer at different pH and concentrations (T = 40 °C).

Solution	Degradation Rate (mg_PBSA_/h/cm^2^)
Sodium phosphate buffer 0.3 M pH 8	6.0
Sodium phosphate buffer 0.5 M pH 8	6.2
Sodium phosphate buffer 0.1 M pH 8	6.4
Sodium phosphate buffer 0.1 M pH 4	5.6
Sodium phosphate buffer 0.1 M pH 6	6.2

## Data Availability

Not applicable.
